# Control of swarming of molecular robots

**DOI:** 10.1038/s41598-018-30187-1

**Published:** 2018-08-06

**Authors:** Jakia Jannat Keya, Arif Md. Rashedul Kabir, Daisuke Inoue, Kazuki Sada, Henry Hess, Akinori Kuzuya, Akira Kakugo

**Affiliations:** 1 Graduate School of Chemical Sciences and Engineering, Hokkaido University, Sapporo, 060-0810 Japan; 2 Faculty of Science, Hokkaido University, Sapporo, 060-0810 Japan; 3 Department of Biomedical Engineering, Columbia University, 1210 Amsterdam Ave., New York, NY 10027 USA; 4 Department of Chemistry and Materials Engineering, Kansai University, Osaka, 564-8680 Japan

## Abstract

Recently we demonstrated swarming of a self-propelled biomolecular motor system microtubule (MT)-kinesin where interactions among thousands of motile MTs were regulated in a highly programmable fashion by using DNA as a processor. However, precise control of this potential system is yet to be achieved to optimize the swarm behavior. In this work, we systematically controlled swarming of MTs on kinesin adhered surface by different physicochemical parameters of MT-kinesin and DNA. Tuning the length of DNA sequences swarming was precisely controlled with thermodynamic and kinetic feasibility. In addition, swarming was regulated using different concentration of DNA crosslinkers. Reversibility of swarming was further controlled by changing the concentration of strand displacement DNA signal allowing dissociation of swarm. The control over the swarm was accompanied by variable stiffness of MTs successfully, providing translational and circular motion. Moreover, the morphology of swarm was also found to be changed not only depending on the stiffness but also body length of MTs. Such detail study of precise control of swarming would provide new insights in developing a promising molecular swarm robotic system with desired functions.

## Introduction

Colonies of bacteria and ants^1–3^, school of fish^4^, or flocks of birds^5,6^ represent typical examples of swarming in nature^7–9^, which emerge through local interactions among the self-propelled living organisms rather than through control by a leader. Swarming offers parallelism, robustness, and flexibility to the organisms^10–12^, and inspired attempts to realize swarming in a synthetic environment using natural^13–15^, chemically powered^16–20^ or mechanical self-propelled objects^21–24^. Self-propelled biomolecular motor systems, such as MT-kinesin, actin-myosin have been particularly promising for realizing such artificial swarming because of their small size, efficiency, and scalability^13,14^. However, a lack of programmability of local interactions among the self-propelled objects prevents us from establishing precise control over the artificial swarming.

To overcome this drawback, we developed a methodology to program local interactions among a large number of MTs driven by biomolecular motor kinesins^25^. By introducing DNA based molecular recognition into the self-propelled system of kinesin driven MTs, we successfully controlled the swarming of thousands of MTs in a reversible fashion. Furthermore, the swarming was programmed using logic gate operations responding to input DNA signals. MT stiffness was shown to be an additional determinant of the swarming behavior. By integrating a photo-responsive compound, azobenzene, to the DNA portion, we have constructed a “molecular swarm robot”, which possesses all the three essential features comprising “robots”, that is the integration of sensors (azobenzene), information processors (DNA), and actuators (MT-kinesin)^26–29^. However, alongside with programming the swarming, understanding the effect of physicochemical parameters of the system is equally important to design complex behavior of molecular swarm robots. To utilize the system with controlled swarm behavior, optimization of the key parameters of the system is essential which has been unexplored yet.

Here, we have systematically explored the role of relevant physicochemical parameters in the reversible regulation of swarming of MTs. We demonstrate how the strength of the DNA interaction regulates the swarming of MTs by varying the concentration of receptor DNA (*r*-DNA), linker DNA (*l*-DNA), dissociation DNA (*d*-DNA). We furthermore investigated how the physical properties of MTs influence the swarming of MTs. A detailed understanding of the physicochemical parameters of different components of the swarming system will enable us to expand the controllability and range of swarming behaviors. The knowledge may advance the development of complex molecular devices utilizing swarming of biomolecular motor systems in a bottom-up manner giving rise to new emergent functions of molecular robots.

## Results

### Swarming of DNA conjugated MTs in a dynamic condition

**Figure 1:**
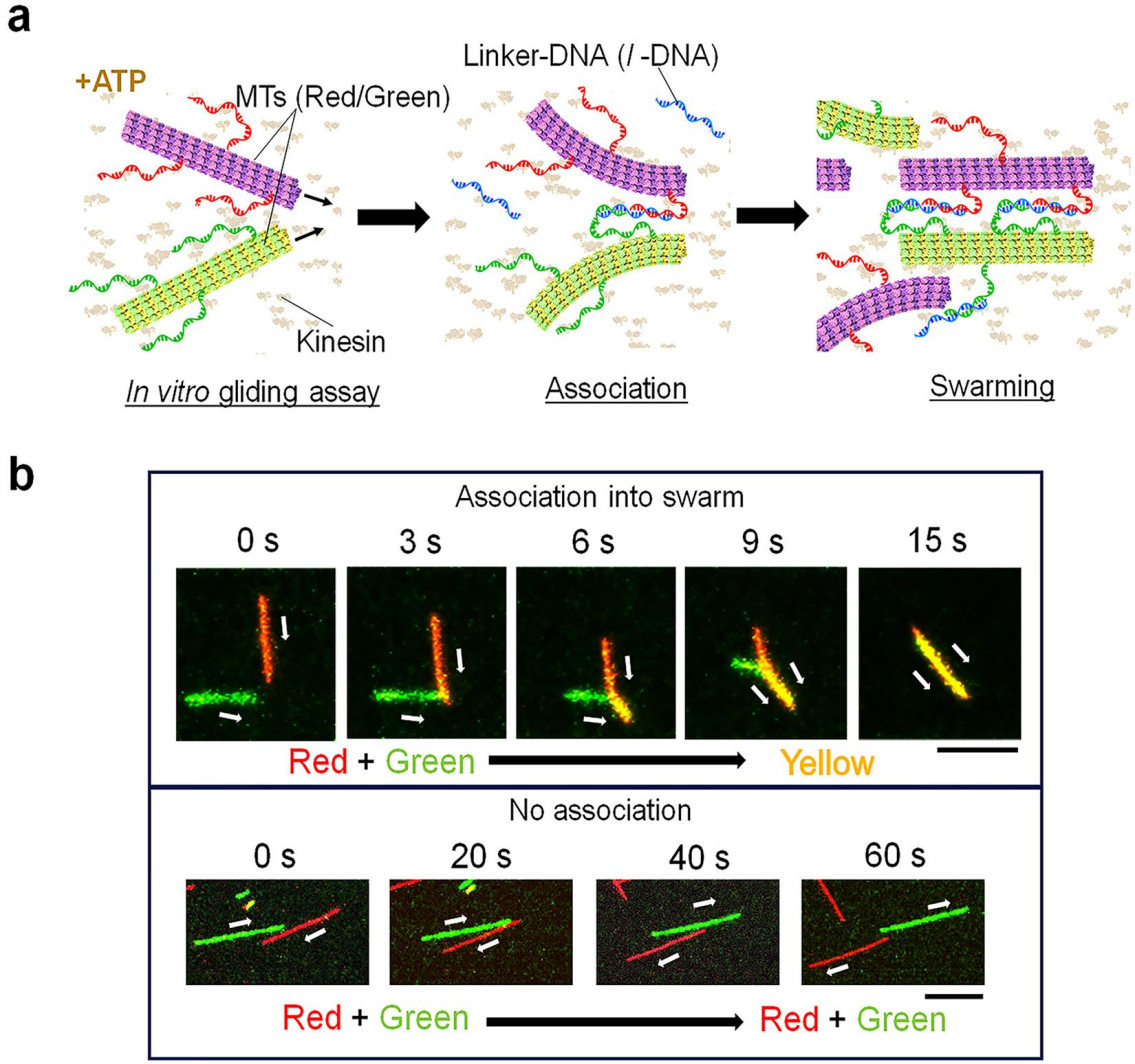
Schematic illustration of the system and interaction between red and green MTs in presence of *l*-DNA. (**a**) MTs are modified with two kinds of fluorescently labeled DNA (*r*-DNAs) and are propelled on a kinesin coated glass slide. Addition of complementary DNA (*l*-DNA) that bridges the two *r*-DNAs triggers swarming of the MTs. (**b**) Upper part: time lapse images of interaction between red and green MTs modified with T_16_ and d(TTG)_5_ respectively in presence of *l*-DNA (d(CAA)_5_A_16_) and move together after swarm formation. Association of red and green MTs exhibits yellow color indicating swarming. Scale bar: 5 μm. Lower part: time lapse images of interaction between red and green MTs in presence of *l*-DNA moving in the opposite direction. Swarming was not observed in this case. Scale bar: 10 μm. Images were captured just after the ATP addition. The concentration of kinesin was 0.3 μM.

Figure 1a shows a schematic illustration of the present molecular swarm system. The individual swarm units were prepared by conjugating MTs and DNA. MTs of lengths between 2 and 10 μm having the path persistence length, *L*_*p*_ of ~580 µm^25^ were conjugated with two different DNA strands, T_16_ and d(TTG)_5_, termed as receptor DNA (*r*-DNA), through a copper-free click reaction. Prior to the conjugation to MTs, T_16_ and d(TTG)_5_ were labeled with a fluorescent dye, TAMRA (red) and FAM (green), respectively in order to allow monitoring of the DNA conjugated MTs under a fluorescence microscope (Supplementary Table S1). The DNA conjugated MTs were propelled by surface-adhered kinesins using the chemical energy of adenosine triphosphate (ATP).

We first investigated how the binding affinity of DNA conjugated MTs promotes swarm formation. We monitored two single MTs conjugated with *r*-DNAs while gliding in the presence of *l*-DNA (d(CAA)_5_A_16_) (Fig. 1b). The *l*-DNA was designed such that it is partially complementary to the *r*-DNAs and may allow crosslinking of the MTs (Supplementary Table S1). Swarming was observed when the red and green MTs collided with each other similar to the snuggling phenomenon^30^. Upon collision, they started to form swarm and moved together with unidirectional motion (Fig. 1b, upper part). According to a previous report such swarm formation by MTs is dependent on collision angle of the MTs^31^. On the other hand, when two MTs approached from totally opposite direction, they passed each other without exhibiting swarming (Fig. 1b, lower part). These results suggest that, swarming of MTs depends on the collision angle of the MTs. The swarm of MTs exhibited translational motion with a velocity (0.52 ± 0.03 μm s^−1^) close to that of single MTs (0.54 ± 0.05 μm s^−1^) which coincides well with our previous work^25^.

Along with the binding interaction of DNA, the driving force of the surface adhered kinesins is another factor that should be considered in the swarming of MTs^32–34^. The motility of the *r*-DNA conjugated MTs was governed by the kinesins which exert force to propel and assemble the MTs into swarms (Supplementary Fig. S1). By varying the concentration of kinesin in the feed (0.001–1 µM), the surface density of kinesin was tuned^35^, which affected the binding of MTs. We have estimated the density of kinesins on substrate for each of the in feed kinesin concentrations as described in the methods section. For the 0.001, 0.01, 0.1, 0.3, 0.5, 0.8, 1 μM in feed concentrations the corresponding kinesin density on the substrate were ~14, 140, 1,400, 4,200, 7,000, 11,200, 14,000 μm^−2^ respectively. At lower concentrations of kinesin (0.001–0.01 µM) at a density 14–140 μm^−2^, the number of MTs bound to the surface was relatively low and motility was rarely observed (Supplementary Fig. S1a). At intermediate concentrations of kinesin (at 0.1–0.3 µM) at a density 1,400–4,200 μm^−2^, the motility and interaction between MTs are facilitated. At higher concentration of kinesin (0.5–1 µM), the binding of MTs to the surface was found to be further increased but the swarming was decreased, which is evident from the decrease in the association ratio (Supplementary Fig. S1b). Therefore, the swarming of MTs is optimally facilitated at a moderate kinesin concentration of 0.3 μM corresponding to the density of ~4,000 μm^−2^ (Supplementary Fig. S1b). We counted the number of individual MTs at initial time and after 30 min of adding ATP buffer and characterized the swarming of MTs by calculating the association ratio (see Methods) at different concentration of kinesin. At 4,000 μm^−2^ density, the swarming was found to increase at a maximum with association ratio of ~70%. Here we estimated the number of kinesins interacting with per unit length of MTs. As already discussed, for the 0.3 μM in feed concentration of kinesin the density was estimated to be ~4,000 μm^−2^, which implies that spacing between two kinesins is ~16 nm. It was reported that kinesin follows a single protofilament of tubulins during its movement along MTs^36^. Considering the width of tubulin as 4.5 nm^37^, we may assume that during motility of MTs on kinesins only one protofilament of tubulins in a MT is able to interact with kinesins. Therefore, the maximum number of kinesins per μm length of a protofilament was estimated to be ~63. This result indicates that for the 0.3 μM in feed concentration of kinesin, there is 1 kinesin for ~2 tubulin dimers along a protofilament of MTs. In this context, our result suggests that the above ratio of kinesin to tubulin dimer is effective to exert optimum driving forces for maximum swarm formation.

### Control of swarming of MTs by designing the length of the *l*-DNA sequence

**Figure 2:**
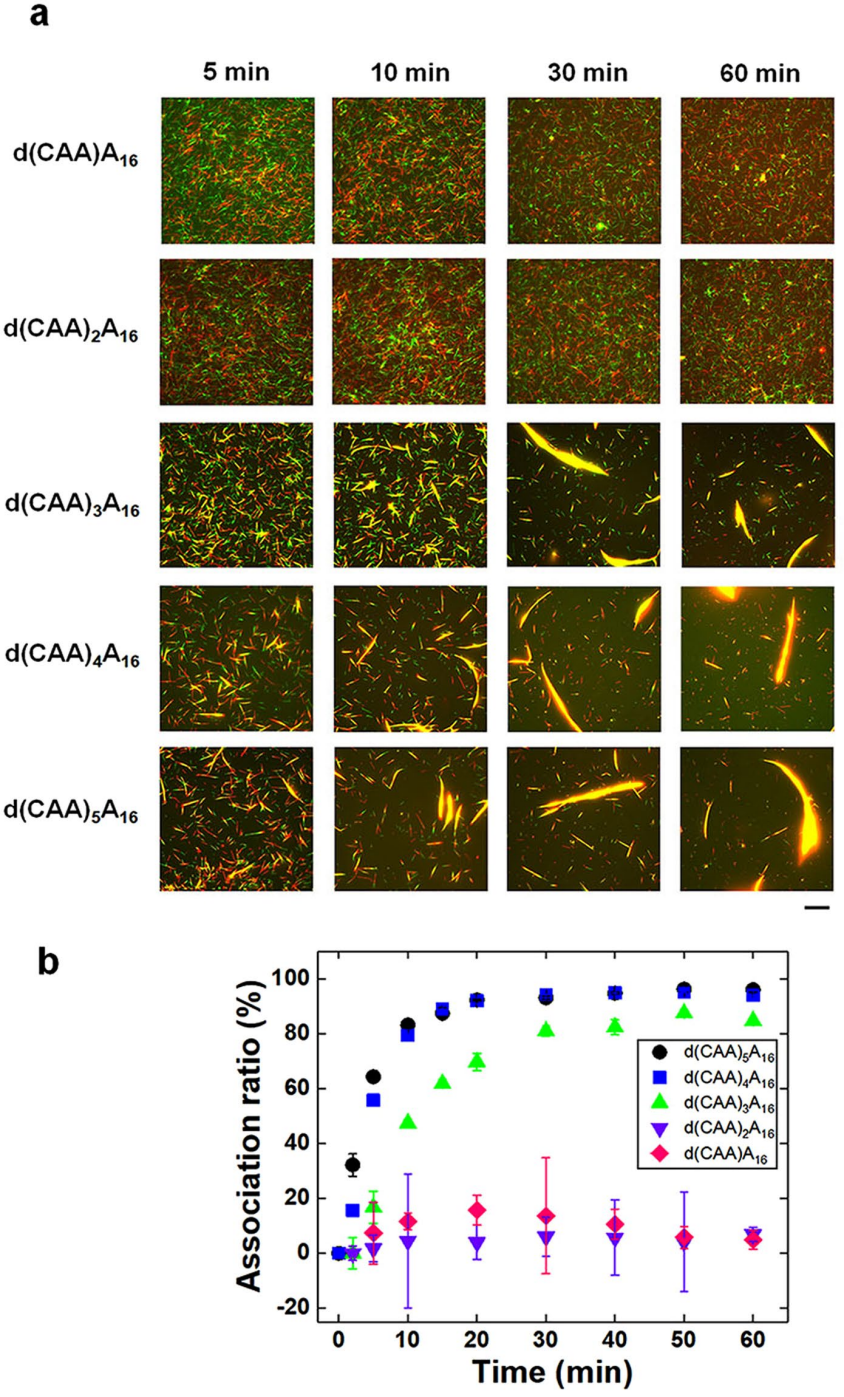
Effect of length of *l*-DNA on the swarming of *r*-DNA1 and *r*-DNA2 conjugated rigid MTs (GMPCPP-MTs). (**a**) Fluorescence microscopy images showing the extent of swarming of rigid MTs upon varying the length of *l*-DNA (d(CAA)A_16_–d(CAA)_5_A_16_). The images were captured after ATP addition and the time was considered 0 min during adding ATP. Scale bar: 20 µm. (**b**) Change in the association ratio of the MTs with time in presence of *l*-DNA with different lengths. The concentration of the *r*-DNA1 and *r*-DNA2 conjugated MTs, i.e. the red and green MTs was 0.6 μM each. The concentration of *l*-DNA for all sequences was fixed at 0.6 μM. The concentration of kinesin was 0.3 μM. Error bar: standard error (s.e.m.).

In order to further confirm that the swarming of the T_16_ and d(TTG)_5_ conjugated MTs is indeed mediated by *l*-DNAs, we have designed *l*-DNAs with different length (d(CAA)_n_A_16_; n = 1–5) (Supplementary Table S2). Simulations predict how the melting temperature (*T*_*m*_) increases with increasing length of *l*-DNA^38^. Depending on the feasibility of hybridization of complementary DNA strands favored by their *T*_*m*,_ the length of *l*-DNA can influence the DNA based self-assembly as found in the previous works^39^. In order to investigate the effect in our system, T_16_ and d(TTG)_5_ conjugated MTs were allowed to move on a kinesin-coated surface at a combined density of ~50,000 mm^−2^. Upon addition of *l*-DNA with CAA repeats shorter than 3, no swarming of MTs was observed (d(CAA)A_16_–d(CAA)_2_A_16_) (Fig. 2a). In the case of *l*-DNA where the number of CAA repeats was 3, the *r*-DNA modified MTs started swarming by interacting with the *l*-DNA (d(CAA)_3_A_16_). While gliding, the red and green MTs came close to each other, associated into swarms appearing yellow in the merged images, and continued moving. For d(CAA)_4_A_16_ (28 base sequences) and d(CAA)_5_A_16_ (31 base sequences), on the other hand, the preferential swarming of the MTs was observed comprising large number of them (Fig. 2a). This may represent that increasing complementary *l*-DNA interaction provided sufficient interaction energy which resulted in the increasing yellow colored swarms of MTs (Fig. 2a). The MTs swarms in dynamic condition are distinguishable from the unstructured aggregates in static condition formed from freely diffusing MTs in absence of kinesin^25^. Along with the thermodynamic feasibility, the swarm formation rate was also influenced by the length of *l*-DNA. As the length of *l*-DNA, i.e. the length of complementary base sequences with *r*-DNA2 was increased, the swarm formation rate accordingly increased, which can be easily understood from the change in their association ratio with time (Fig. 2b). For d(CAA)_4_A_16_ and d(CAA)_5_A_16_, the swarm formation rate was found maximum up to ~95–98% after reaching a plateau with time.

### Effect of the concentration of *r*-DNAs and *l*-DNA on swarming of MTs

As demonstrated above, the swarming of MTs relies on the interaction between *r*-DNAs and *l*-DNA. So, a systematic investigation was performed to determine how the concentration of the *r*-DNAs and *l*-DNA affects the swarming. In the investigation, an almost equal density of red and green MTs (1:1 density ratio) was used in the experiments at which swarms with highest association ratio and largest size were formed (Supplementary Fig. S2).

**Figure 3:**
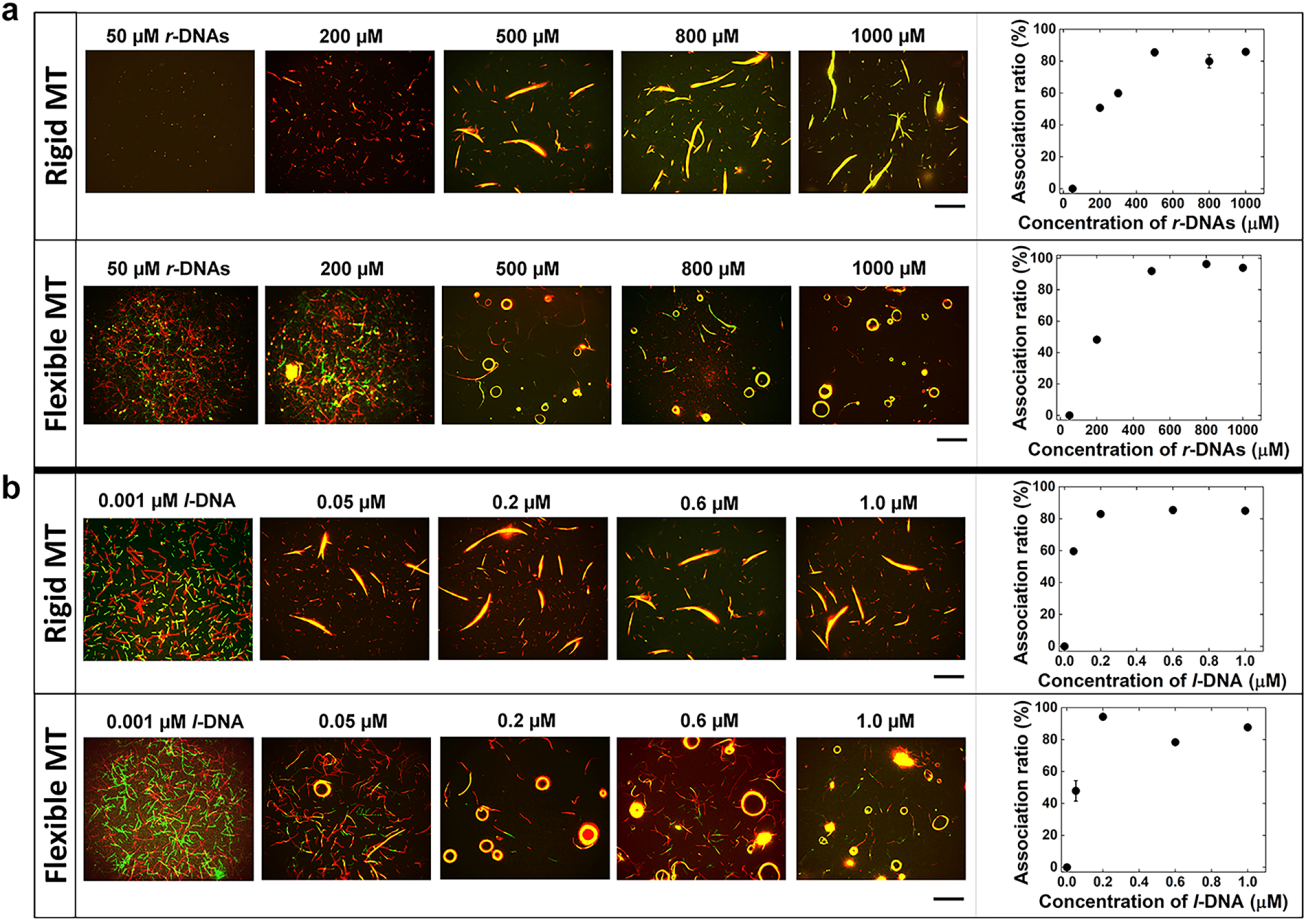
Effect of the concentration of *r*-DNAs (*r*-DNA1 and *r*-DNA2) in the feed, used to conjugate to the rigid MTs (GMPCPP-MTs) and flexible MTs (GTP-MTs), and concentration of *l*-DNA on the swarming of the MTs. (**a**) Fluorescence microscopy images of the swarms of rigid and flexible MTs with translational and circular motion respectively formed upon varying the concentrations of *r*-DNA1 (for red MTs) and *r*-DNA2 (for green MTs) in the feed. The graphs show the change in association ratio upon changing the concentration of *r*-DNAs (50–1,000 µM). The concentration of *l*-DNA was 0.6 μM. (**b**) Fluorescence microscopy images of the swarms of rigid MTs and flexible MTs formed by varying the concentration of *l*-DNA (0.001–1 µM) and their corresponding change in association ratio. The concentration of *r*-DNAs in the feed was 500 µM each. (rigid and flexible MTs). The images were captured after 60 min of ATP addition (a) and (b). The concentration of the *r*-DNA1 and *r*-DNA2 conjugated MTs was 0.6 μM each. The concentration of kinesin was 0.3 μM. Scale bar: 50 µm. Error bar: s.e.m.

We first investigated the effect of the concentration of *r*-DNAs on swarming of MTs. Fixing the concentration of *l*-DNA at 0.6 µM, we varied the concentration of *r*-DNAs (T_16_ and d(TTG)_5_) used for MT modification from 50–1,000 µM. The labeling ratio of *r*-DNA to MTs i.e., tubulin dimers was found to increase as the concentration of *r*-DNAs increased, and reached ~100% when the concentration of *r*-DNA was 500 µM^25^. Although MTs exhibited swarming with translational motion when the concentration of *r*-DNA was 200 µM, more swarming was observed at 500 µM of *r*-DNA, which is evident from the corresponding association ratio (Fig. 3a, rigid MTs) approximately ~87% within 60 min after the addition of *l*-DNA. Further increase in *r*-DNAs concentration (800–1,000 µM) brought no considerable change in the association ratio (Fig. 3a). By changing polymerization condition (see experimental section), we changed the rigidity of MTs and found that more MTs exhibited swarming with circular motion upon decreasing their rigidity with *L*_*p*_ of ~245 µm (Fig. 3a, flexible MTs)^25^. Even though the rigidity of MTs induced such a change in swarming mode, no difference in the effect of concentration of *r*-DNA was observed for the two systems. At 500 µM of *r*-DNA, increase in circular swarming was observed which reached a plateau at 800 µM–1,000 µM *r*-DNA concentration with association ratio of ~92–95%. Similar trend in association ratio change was obtained as in the case of rigid MTs. Based on these outcomes, a phase diagram was constructed correlating the concentration of *r*-DNAs with the swarming of MTs (Supplementary Fig. S3a).

We then investigated the effect of the concentration of *l*-DNA on swarming of MTs. While the concentration of *r*-DNA1 and *r*-DNA2 was fixed at 500 µM, the concentration of *l*-DNA (d(CAA)_5_A_16_) was varied from 0.001 to 1 µM (Fig. 3b). At low concentration of *l*-DNA (0.001 µM), swarming was observed for neither the rigid nor flexible MTs. Swarming was only observed when the concentration of *l*-DNA was above 0.05 µM. As the concentration of *l*-DNA was further increased (0.2–1 µM), the association ratio initially increased and then reached a plateau for both, rigid and flexible MTs with approximate 88% association ratio. The effect of *l*-DNA concentration on the swarming of MTs is again summarized in a phase diagram (Supplementary Fig. S3b).

### Design of *d*-DNA and effect of its concentration on the reversible swarming

**Figure 4:**
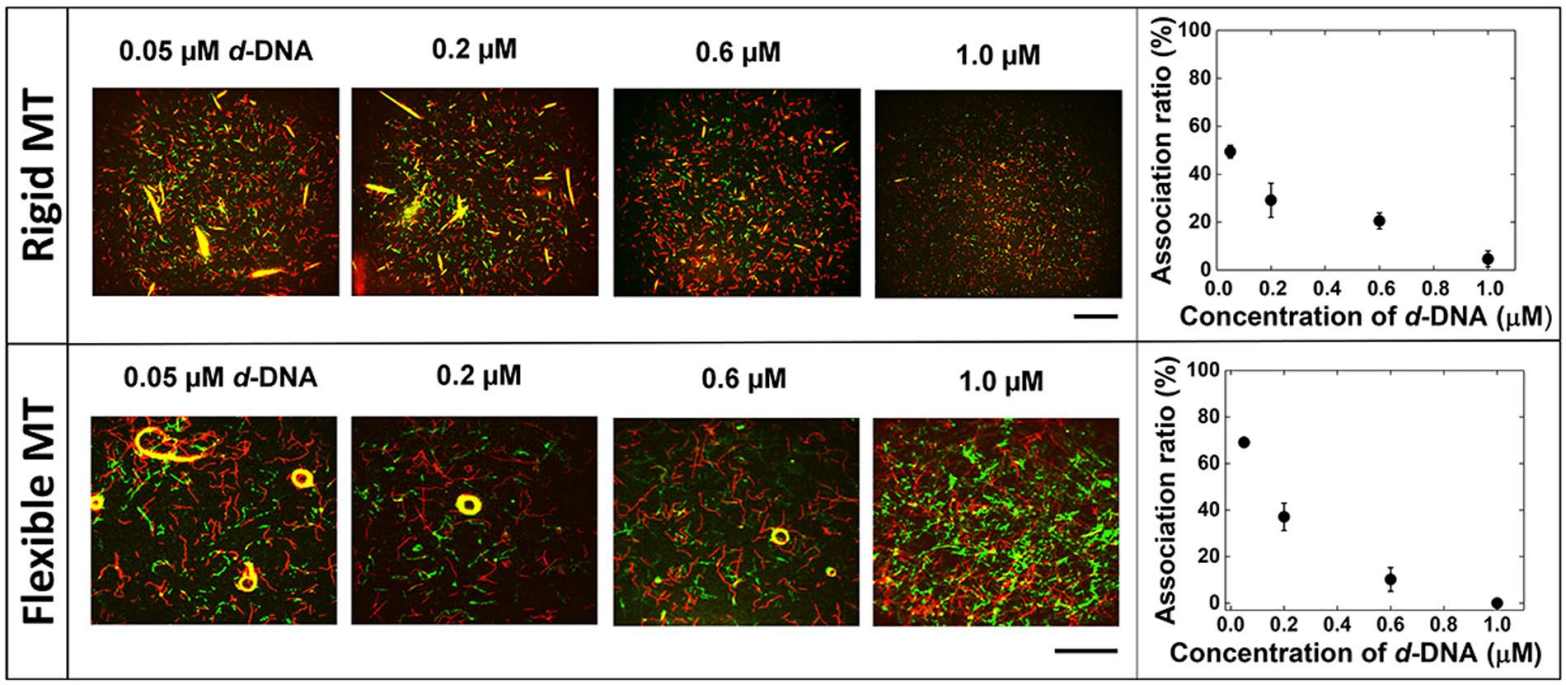
Effect of the concentration of *d*-DNA on the dissociation of the swarms of *r*-DNA1 and *r*-DNA2 conjugated rigid MTs (GMPCPP-MTs) and flexible MTs (GTP-MTs) into single MTs. Fluorescence microscopy images showing the rigid MTs after dissociation of the swarms with translational motion in the presence of varying concentrations of *d*-DNA (0.05–1.0 µM); the graph shows the effect of the concentration of *d*-DNA on the association ratio of the MTs (Rigid MT). Fluorescence microscopy images showing the flexible MTs after dissociation of the swarms with circular motion and association ratio measured for swarming with circular motion upon varying the concentration of *d*-DNA (Flexible MT). The *d*-DNA was introduced after 60 min of ATP addition in order to allow the formation of swarms in the presence of *l*-DNA (0.6 μM). The images were captured and the association ratio was measured after 60 min of *d*-DNA addition. Scale bar: 50 µm. The concentration of the *r*-DNA1 and *r*-DNA2 conjugated MTs, i.e. the red and green MTs was 0.6 μM each. The concentration of kinesin was 0.3 μM. Error bar: s.e.m.

DNA strand displacement reactions, in which a DNA strand is displaced by another one, have been proved useful in controlling self-assembly based on DNA interactions^40–43^. Therefore, we designed a DNA sequence (*d*-DNA) such that it facilitates dissociation of the swarms into isolated MTs. Strand displacement requires that the binding energy of *d*-DNA with *l*-DNA is higher than that between *l*-DNA and *r*-DNAs (Supplementary Table S1) which is easily predictable from their *T*_*m*_^25,38^. A DNA sequence (*d*-DNA), which was fully complementary to the *l*-DNA sequence was chosen to withdraw the *l*-DNA from MT swarms by the DNA strand displacement reaction. Upon addition of *d*-DNA (0.05 µM) swarms of MTs with translational and circular motion began to dissociate into single MTs, which was quantified by a significant decrease in the association ratio (Fig. 4). A further increase in the concentration of *d*-DNA decreased the association ratio further. At 1 µM *d*-DNA, the association ratio was close to zero and the MT swarms were almost completely dissociated (Fig. 4).

### Effect of the length of MTs on swarming mode

**Figure 5:**
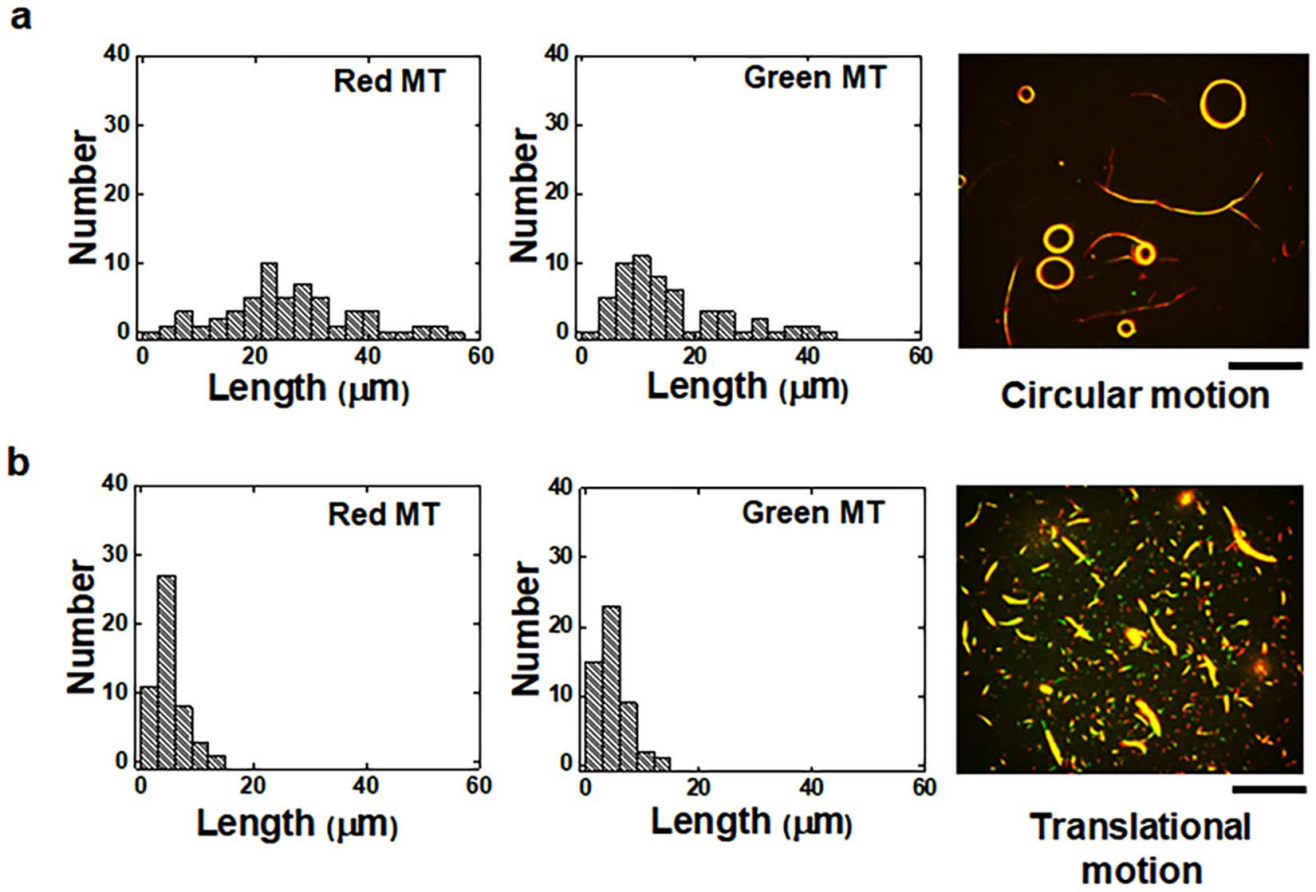
Effect of the length of *r*-DNA1 and *r*-DNA2 conjugated flexible MTs on the mode of swarming of the MTs. (**a**) Histograms of the length of *r*-DNA1 conjugated MTs (red MTs) and *r*-DNA2 conjugated MTs (green MTs) just after their preparation. The fluorescence microscopy image shows the swarms of MTs which exhibited circular motion. The average lengths of the red and green flexible MTs were 22.2 ± 12.5 µm and 16.2 ± 9.0 µm respectively. (**b**) Histograms of the length of the *r*-DNA1 conjugated MTs (red MTs) and the *r*-DNA2 conjugated MTs (green MTs) after shear treatment (ten times). The fluorescence microscopy image shows the swarms of MTs which exhibited translational motion. The length of the red and green flexible MTs was 4.7 ± 2.5 µm and 4.7 ± 2.4 µm respectively. The number of MTs considered for the length measurement was 50 in each case. The images were captured after 60 min of the addition of *l*-DNA and ATP. The concentration of the red and green MTs was 0.6 μM each. The concentration of *l*-DNA was 0.6 μM. The concentration of kinesin was 0.3 μM. Scale bar: 50 µm.

We previously demonstrated that the rigidity of MTs can be utilized to control the swarming mode of MTs. An open question is if the length of MTs has any influence on their swarming mode. Flexible MTs with different lengths were prepared by applying shear stress using a micro syringe as reported previously^44,45^. The length of MTs was tuned by varying the number of shear treatments (up to ten times). The average lengths of flexible red and green MTs were 22.2 ± 12.5 µm (average ± standard deviation (s.d.)) and 16.2 ± 9.0 µm respectively just after preparation (Fig. 5a). After ten shear treatments the length of red and green MTs were reduced to 4.7 ± 2.5 µm and 4.7 ± 2.4 µm respectively (Fig. 5b). The changes in the average length of the flexible MTs due to the shear treatment match the results reported in previous studies^44,45^. When the shortened MTs were employed in swarming, the swarming mode was found to change from circular to translational one (Fig. 5a,b). This result indicates that the MT length plays an important role in determining the morphology of MT swarms.

## Discussion

In order to develop a robustly controlled molecular swarm system, we systematically investigated the effect of related physicochemical parameters on the DNA assisted swarming of a self-propelled biomolecular motor system, MT-kinesin. We found that the effects are comparable to those discovered in previous studies where self-assembly of MTs was regulated using streptavidin (St)-biotin (Bt) crosslinkers system^32–34^. Unlike the self-assembly of MTs using the strong St-Bt interaction, where MTs assembled even when they moved in opposite direction^32,33^, almost no sticking of MT swarms on the kinesin-coated surface was noticed in the present system. This observation suggests that swarming of MTs was favored by moderate interaction between *l*-DNA and *r*-DNAs attached to MTs. The *r*-DNAs and *l*-DNA are designed such that they can bind in an unzipping manner, a feasible model for DNA interaction^46^. DNA hybridization through the unzipping mode was further facilitated when the MTs collided while they were moving in the same direction just before the collision. Consequently, MTs started to form swarm due to DNA hybridization through the unzipping mode. However, for the MTs that collided while moving in the opposite direction, no successful swarm formation was observed. A similar phenomenon was previously reported in which MTs moving in the opposite direction in a crowded condition also failed to form stable bundles^30^. The failure in swarm formation by the MTs could be ascribed to the direction of force exerted by the kinesins to the MTs moving in the opposite direction, and energetics of the associated DNA hybridization, confirmation of which requires further investigation.

In addition to that, our study is helpful in controlling the swarm system more efficiently by tuning the interaction not only depending on the concentration of the components but also by designing properties of the crosslinkers such as the length of *l*-DNA sequence. This was previously done to tune the self-assembly of particle systems in static conditions^39^. Here, we tuned the binding interactions of MTs under dynamic conditions by optimizing the sequence of *l*-DNA (Fig. 2). The contributions of the concentrations of *r*-DNA, *l*-DNA, *d*-DNA, and kinesin, and the physical properties of MTs were also found to have a profound influence on the swarming of MTs. Nearly complete association was obtained with DNA constituents above certain concentrations (Fig. 3). The phase diagrams predicted from the results indicate similar effects to those found in previous work^34^ (Supplementary Fig. S3). The reverse control responding to the *d*-DNA is favored by their elevated *T*_*m*_ as predicted in the equilibrium condition^25^, which was also successfully tuned by varying the concentration of *d*-DNA. By selective parameter combinations, we are able to regulate not only the thermodynamics and kinetics of MT swarming (Fig. 2) but also the morphology of MT swarms between translational and circular modes corresponding to their stiffness and body length (Fig. 5).

We expect that the present study will enable the precise designing of DNA assisted swarming of self-propelled biomolecular motor systems^47^. Moreover, our observations point towards a way of greatly improving the selectivity of DNA detection systems based upon biomolecular motor systems and also various applications such as active DNA sensors, adaptive actuators or analyte concentrators by optimization of these systems. However, apart from the chemical signal of DNA, physical signal by using light sensor has further advancements such as non-invasiveness, spatiotemporal regularity which are lacking in the present work. Insertion of a light sensor molecule in the system^25^, could provide spatiotemporal control over swarming of MTs in a non-invasive way. Varying different parameters of light sensors such as number of photoresponsive azobenzene molecules in the DNA sequences, intensity of light, regulating localized association and dissociation of MTs swarms responding to the light signal; spatiotemporal control over molecular swarm robots could be further achieved which is expected to present as a future prospect. The knowledge from present and future work would expand potential applications of biomolecular motors with precisely controlled computation and may ultimately benefit molecular robotics^26,27,48^.

## Methods

### Purification of tubulin and kinesin

Tubulin was purified from porcine brain using a high-concentration PIPES buffer (1 M PIPES, 20 mM EGTA, and 10 mM MgCl_2_) and stored in BRB80 buffer (80 mM PIPES, 1 mM EGTA, 2 mM MgCl_2_, pH adjusted to 6.8 using KOH)^49^. Recombinant kinesin-1 consisting of the first 573 amino acid residues of human kinesin-1 was prepared as described in the literature^50^. Azide labeled tubulin was prepared using N3-PEG4-NHS following the established protocol of labeling tubulin with fluorescent dye^51^. The tubulin concentration was determined by measuring the absorbance at 280 nm using a UV spectrophotometer (Nanodrop 2000c).

### Design and preparation of DNA sequences

*r*-DNA and *l*-DNA strands were designed by calculating the melting temperature of different sequences using ‘OligoAnalyzer 3.1’ software^52^, and selecting sequences with melting temperatures between 0 °C and 50 °C for experimental testing. Dibenzocyclooctyne (DBCO) and fluorescence dye labeled strands were chemically synthesized using appropriate CPG columns and a phosphoramidite monomer (Glen Research, VA) on an ABI 3900 automatic DNA synthesizer, purified by reverse phase HPLC and fully characterized by MALDI-TOF/MS (Bruker microflex LRF). The *r*-DNA was modified at the 3′ end with either 5(6)-carboxytetramethylrhodamine (TAMRA) or 5-carboxyfluorescein (FAM) and at the 5′ end with DBCO. *l*-DNAs and *d*-DNA were purchased from Eurofins Genomics LLC.

### Preparation of MTs

MTs were prepared by adding azide-tubulin to polymerization buffer (80 mM PIPES, 1 mM EGTA, 1 mM MgCl_2_, 1 mM polymerizing agent, pH~6.8) to a final concentration of 56 µM tubulin and incubating at 37 °C for 30 min. The polymerizing agent for flexible MTs was guanosine triphosphate (GTP), and the polymerizing agent for rigid MTs was guanylyl-[(α, β)-methyleno] diphosphonate (GMPCPP), a slowly hydrolyzable analogue of GTP. Dimethyl sulfoxide (DMSO) was added to a final concentration of 5% for the polymerization of flexible MTs, but not for rigid MTs. A copper free click reaction was initiated by adding 3.5 µL DBCO conjugated *r*-DNAs (500 µM) to the 5 µL azide-MTs (56 µM), which allowed an azide-alkyne cycloaddition reaction, and incubated the solution at 37 °C for 6 hours^53^. 100 µL of cushion buffer (BRB80 buffer supplemented with 60% glycerol) was used to separate the MTs by centrifugation at 201,000 g (S55A2–0156 rotor, Hitachi) for 1 hour at 37 °C. After removing the supernatant, the pellet of *r*-DNA conjugated MTs was washed once with 100 µL BRB80P (BRB80 supplemented with 1 mM taxol) and dissolved in 15 µL BRB80P.

### Flow cells and motility assays for the demonstration of swarming of MTs

A flow cell with dimensions of 9 × 2.5 × 0.45 mm^3^ (L × W × H) was assembled from two cover glasses (MATSUNAMI Inc.) using double-sided tape as spacer. The flow cell was filled with 5 μL casein buffer (BRB80 buffer supplemented with 0.5 mg mL^−1^ casein). After incubating for 3 min, 0.3 μM kinesin solution was introduced into the flow cell and incubated for 5 min resulting in a kinesin density of 4,000 μm^−2^ on the substrate^35^. After washing the flow cell with 5 µL of wash buffer (BRB80 buffer supplemented with 1 mM DTT, 10 μM taxol), 5 µL of red MTs (TAMRA labeled *r*-DNA1 MTs) solution were introduced and incubated for 2 min, followed by washing with 10 µL of wash buffer. Subsequently, 5 µL of green MTs solution were introduced and incubated for 2 min, followed by washing with 10 µL of wash buffer. The green MTs (FAM labeled *r*-DNA2 MTs) were incubated with *l*-DNA for 15 min at room temperature prior to addition into flow cell. The motility of red and green MTs was initiated by applying 5 µL of ATP buffer (wash buffer supplemented with 5 mM ATP, 4.5 mg mL^−1^ D-glucose, 50 U mL^−1^ glucose oxidase, 50 U mL^−1^ catalase and 0.2% methylcellulose (w/v)). The time of ATP addition was set as 0 min. Soon after the addition of ATP buffer the flow cell was placed in an inert chamber system (ICS)^54^ and the MTs were monitored using an epi-fluorescence microscope at room temperature (25 °C). The experiments were performed at least 3 times for each condition.

### Tuning the length of MTs

MTs with different lengths were prepared by applying shear stress, as reported previously^44,45^, to DNA conjugated MTs using a micro syringe (Hamilton syringe with an inner diameter of 2.06 mm) and a PEEK tube (length: 50 mm, nominal inner diameter: 0.26 mm). 30 μL of MT solution (0.6 μM) were passed back-and-forth through the syringe-mounted PEEK tube by manual operation of the syringe. The length of flexible MTs (GTP-MTs) was tuned by varying the number of passages (up to ten times). The change in length of GTP-MTs before and after shear treatment was manually measured using the image analysis software ‘ImageJ’. The average lengths of red and green GTP-MTs were 22.2 ± 12.5 µm and 16.2 ± 9.0 µm respectively just after preparation. After ten shear treatments, the length of red and green GTP-MTs was reduced to 4.7 ± 2.5 µm and 4.7 ± 2.4 µm respectively. The changes in the average length of the GTP-MTs due to the shear treatment coincide well with the results reported in previous studies^44,45^.

### Estimation of kinesin density

Kinesin density was estimated following a previous report where density of kinesin on a substrate surface was estimated using quartz crystal microbalance (QCM)^35^. A standard curve was prepared using the previously reported data from which the following relationship between in feed concentration of kinesin and kinesin density on a substrate was obtained: y = 1.4 ∗ 10^4^x, where x and y are kinesin concentration in feed (μM) and kinesin density on the substrate (μm^−2^) respectively. Using this relationship, we then estimated the kinesin density for each of the in feed concentration of kinesin employed in this study.

### Fluorescence microscopy

The samples were illuminated with a 100 W mercury lamp and visualized by an epifluorescence microscope (Eclipse Ti, Nikon) using an oil-coupled Plan Apo 60× N.A.1.4 objective (Nikon). UV cut-off filter blocks (TRITC: EX 540/25, DM565, BA605/55; GFP-B: EX460-500, DM505, BA510-560; Nikon) were used in the optical path of the microscope. Images were captured using a cooled-CMOS camera (NEO sCMOS, Andor) connected to a PC. Two ND filters (ND4, 25% transmittance for TRITC and ND1, 100% transmittance for GFP-B) were inserted into the illumination light path of the fluorescence microscope to reduce photobleaching of the samples.

### Image analysis and measurement of the association ratio of MTs

The length of MTs was measured from images captured by fluorescence microscopy using image analysis software (ImageJ).

The association ratio at a given time *t* was determined by counting the number of single MTs manually and dividing the number at time *t* by the number present initially (*t* = 0). The time-dependent association ratio *R(t)* of red and green MTs was determined as follows

1 $$R(t)=\frac{N(0)-N(t)}{N(0)}$$

with

*N* (0) = Initial number of single MTs,

*N* (*t*) = Number of single MTs after time *t*.

The mean association ratio was obtained from the average of four regions of interest (126.5 μm × 126.5 μm).

### Data availability

The data that support this study are available from the corresponding author upon reasonable request.

## Electronic supplementary material

Supplementary Information
