# Supplementary material for: Control of swarming of molecular robots
Source: Sci Rep. 2018 Aug 6;8:11756. doi: 10.1038/s41598-018-30187-1 (PMC6079095; doi:10.1038/s41598-018-30187-1)
Supplement: Supplementary file 1 — Supplementary Information [file 41598_2018_30187_MOESM1_ESM.docx]

**Control of swarming of molecular robots**

Jakia Jannat Keya, Arif Md. Rashedul Kabir, Daisuke Inoue, Kazuki Sada, Henry Hess,

Akinori Kuzuya, * and Akira Kakugo *

**Supplementary Figures**

**
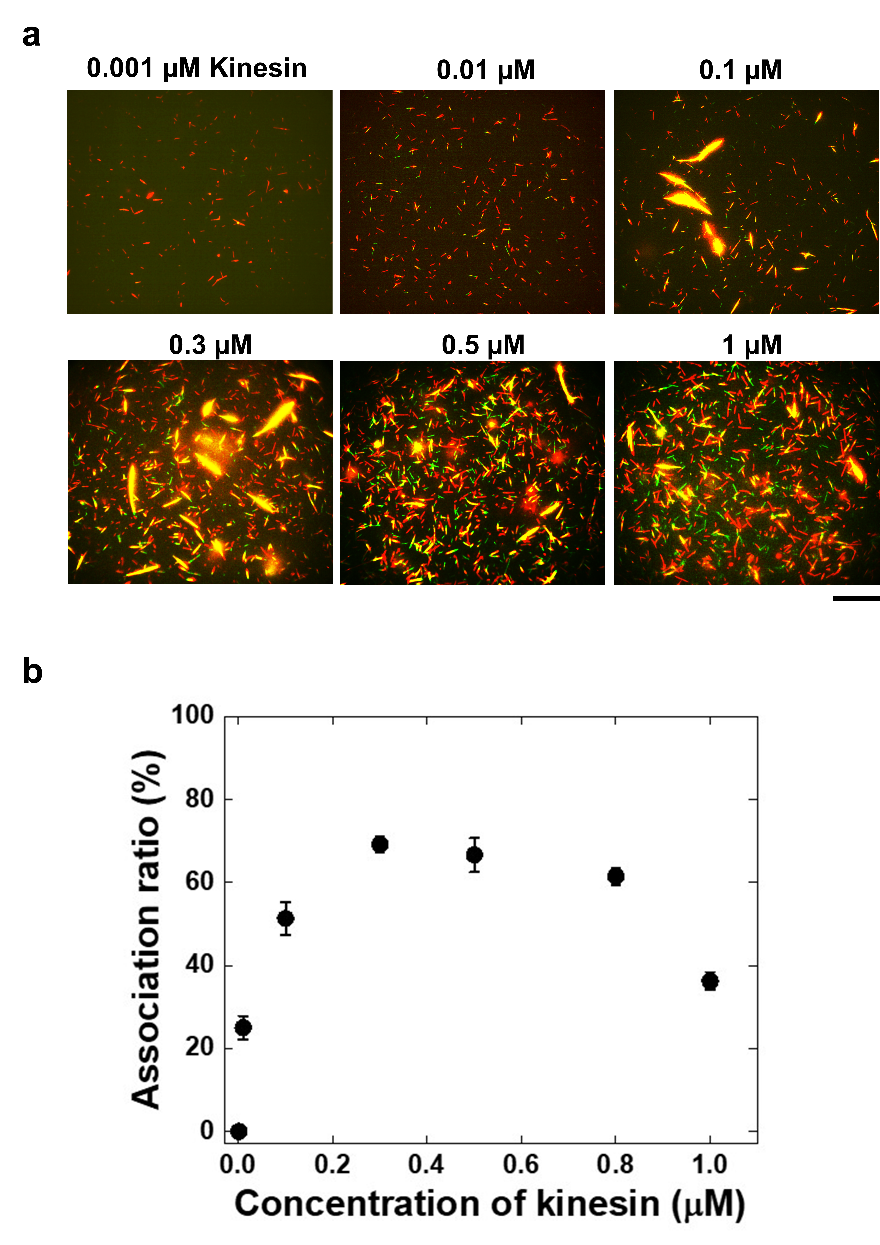
**

**Figure S1. Effect of kinesin concentration in the feed on the swarming of *r*-DNA1 and *r*-DNA2 conjugated MTs.** (**a**) Fluorescence microscopy images showing the effect of kinesin concentration (0.001–1 μM) in the feed on the swarming of MTs. The images were captured after 30 min of ATP addition. Scale bar: 50 µm. (**b**) MT association ratio as a function of the kinesin concentration in the feed. The concentration of both *r*-DNA1 and *r*-DNA2 conjugated MTs (red and green MTs respectively) was ~0.6 μM, the concentration of *l*-DNA was 0.6 μM in all cases. Error bar: standard error (s.e.m.).

**
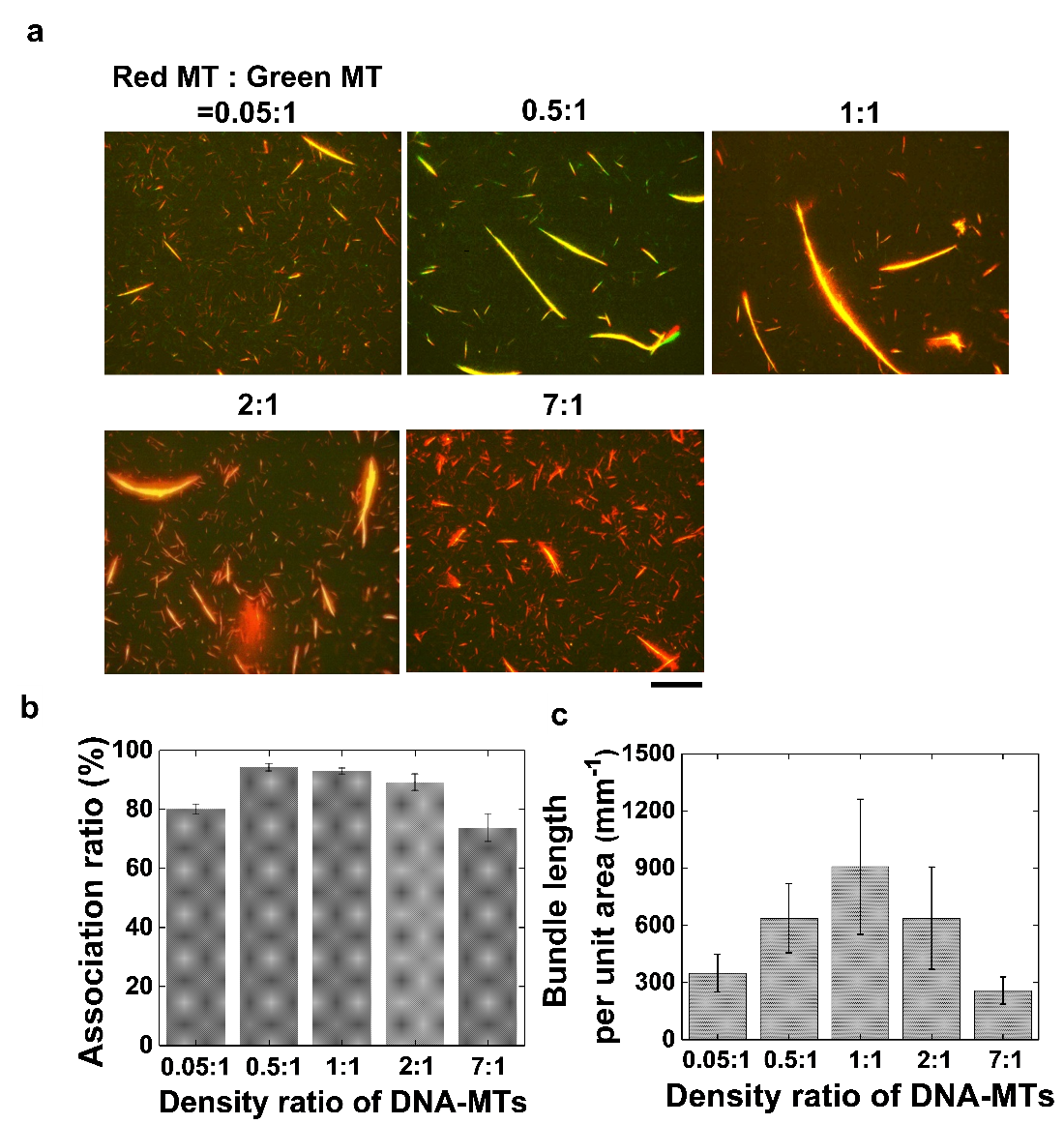
**

**Figure S2. Effect of the density ratio of *r*-DNA1 and *r*-DNA2 conjugated rigid MTs (GMPCPP-MTs) on the swarming of the MTs.** (**a**) Fluorescence microscopy images showing the swarms of MTs formed when the density ratio between *r*-DNA1 and *r*-DNA2 conjugated MTs was changed (*r*-DNA1 MT:*r*-DNA2 MT= 0.05:1, 0.5:1, 1:1, 2:1, 7:1). Scale bar: 50 µm. The images were captured after 60 min of ATP addition. (**b**) Effect of the density ratio of *r*-DNA1 and *r*-DNA2 conjugated MTs on the MT association ratio. (**c**) Effect of the density ratio of *r*-DNA1 and *r*-DNA2 conjugated MTs on the bundle length (mm) per unit area (mm^2^). The concentration of the *r*-DNA2 MTs (green MTs) in the feed was fixed at 0.6 μM and that of the *r*-DNA1 modified MTs (red MTs) was varied from 0.18 μM, over 0.3 μM, 0.6 μM, 1.2 μM, to 4.2 μM to obtain density ratios of 0.05:1, 0.5:1, 1:1, 2:1, 7:1 respectively. The concentration of *l*-DNA was fixed at 0.6 μM in all cases. The concentration of kinesin was 0.3 μM. Error bar: s.e.m.


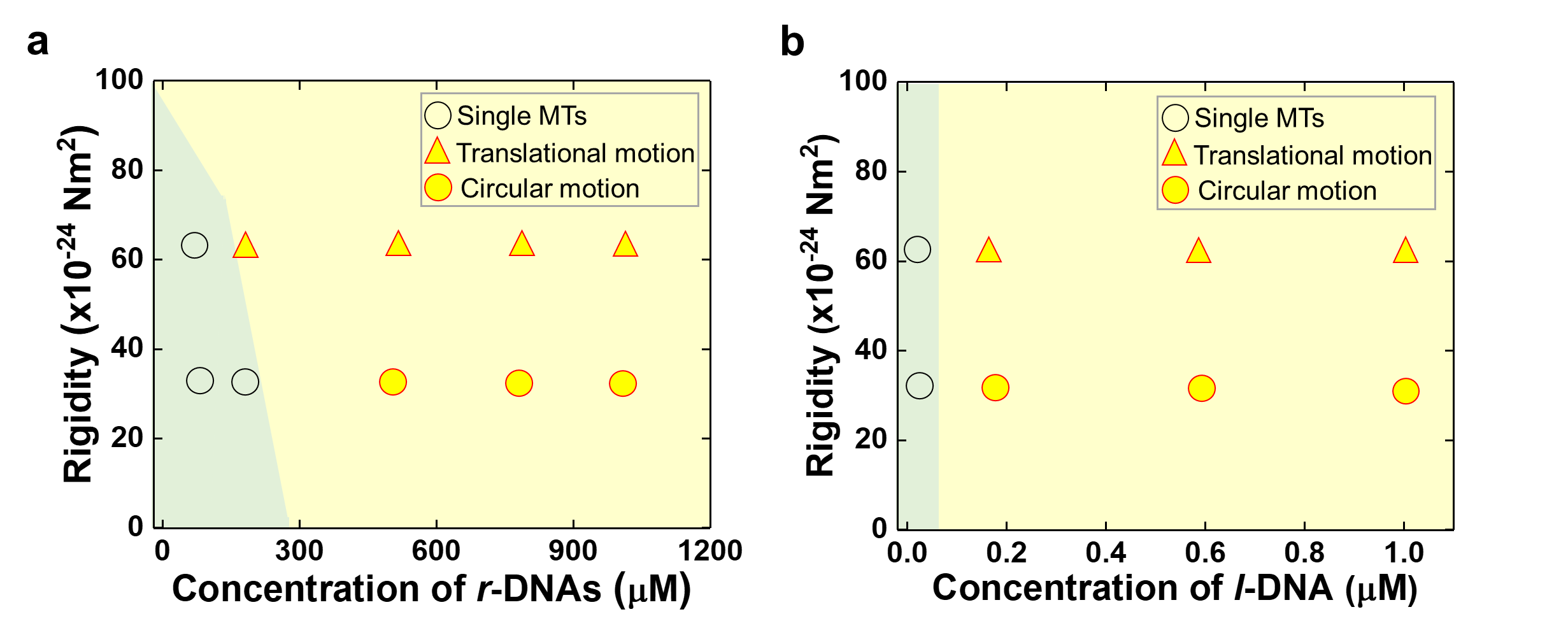


**Figure S3.** **Phase diagrams show the evolution of different swarming modes of MTs with the change of the rigidity of MTs and concentration of *r*-DNAs and *l*-DNA.** (**a**) A phase diagram showing the effect of the rigidity of MTs and the concentration of *r*-DNAs on the swarming mode of the MTs. The concentration of *l*-DNA was fixed at 0.6 μM. (**b**) A phase diagram showing the effect of the rigidity of MTs and concentration of *l*-DNA on the swarming mode of the MTs. The concentration of *r*-DNA1 and *r*-DNA2 was fixed at 500 µM maintaining the labeling ratio of ~100% of *r*-DNAs to MTs. The concentration of red and green MTs in the feed was 0.6 μM. The concentration of kinesin was 0.3 μM. Rigidity values used for GTP-MTs and GMPCPP-MTs were 34×10^-24^ Nm^2^ and 62×10^-24^ Nm^2^ respectively.^1^

**Supplementary Tables**

**Table S1.** The sequences of *r*-DNAs, *l*-DNA and *d*-DNA used to demonstrate the swarming of MTs and dissociation of the swarms.

| **DNA** | **Sequence (5´-3´)** | **5´ end** | **3´ end** |
| --- | --- | --- | --- |
| *r*-DNA1 | TTTTTTTTTTTTTTTT | DBCO | TAMRA |
| *r*-DNA2 | TTGTTGTTGTTGTTG | DBCO | FAM |
| *l*-DNA | CAACAACAACAACAAAAAAAAAAAAAAAAAA | - | - |
| *d*-DNA | TTTTTTTTTTTTTTTTGTTGTTGTTGTTGTT | - | - |

**Table S2.** The sequences of the *l*-DNA with different length.

| **DNA** | **Sequence (5´-3´)** | **5´ end** | **3´ end** |
| --- | --- | --- | --- |
| d(CAA)A_16_ | CAAAAAAAAAAAAAAAAAA | - | - |
| d(CAA)_2_A_16_ | CAACAAAAAAAAAAAAAAAAAA | - | - |
| d(CAA)_3_A_16_ | CAACAACAAAAAAAAAAAAAAAAAA | - | - |
| d(CAA)_4_A_16_ | CAACAACAACAAAAAAAAAAAAAAAAAA | - | - |
| d(CAA)_5_A_16_ | CAACAACAACAACAAAAAAAAAAAAAAAAAA | - | - |

**Supplementary Reference**

1. Mickey, B. & Howard, J. Rigidity of microtubules is increased by stabilizing agents. *J. Cell Biol.* **130,** 909-917 (1995).
